# Supplementary material for: Determinants of testing for HIV among young people in Uganda. A nested, explanatory-sequential study
Source: PLOS Glob Public Health. 2022 Dec 5;2(12):e0000870. doi: 10.1371/journal.pgph.0000870 (PMC10022384; doi:10.1371/journal.pgph.0000870)
Supplement: S1 File — (DOCX) [file pgph.0000870.s001.docx]

In-depth Interview Guide for young people.

**Introduction**

We are part of a research team from the Makerere University College of Health Sciences. We are studying the proportion of HIV testing among young people, factors associated, barriers, and facilitators of HIV testing among young people in Wakiso District.

You have been selected for the second part of the study because we are interested in youth experiences with regard to HIV testing in Wakiso district. Your participation will be voluntary.

I would like to request your permission to record our conversation so that I can remember it more easily later. Only the research team will have access to the interview recordings. If we share anything you said, we will remove your name and other identifying information to protect your privacy. After the study, all recordings will be discarded /deleted.

If there is any question you don’t want to answer, you don’t have to answer it.

Is it ok if I turn on the recorder? [Pause]

Thank you.

S/county…………………… Parish…………………Venue…………………………

Date……………… Starting time…………… ending time…………

1. Please tell me about your interests? Please tell me about yourself
2. What do you know about HIV/AIDS? What have you heard about the way it is transmitted?
3. What have you heard about HIV testing? Where did you hear that from and from whom?
4. If you have ever tested for HIV, how did you come to this decision? Whom did you go with for testing?

If you have never tested for HIV, what reasons do you have for not testing? Have you had any attempts to test? Why did they fail?

1. Where in this area can people go if they want to test for HIV? Which sites? How did you hear about them? What are they like? Do people talk about them? What do they say?
2. What do you think about HIV testing? Why is testing for HIV important?
3. What benefits does someone get from knowing his/her HIV status? If Positive? If Negative?
4. What challenges have you faced that stopped or delayed you from having to test for HIV?
5. What do young people generally think about HIV testing? What about your friends**?**
6. What does your family think about HIV testing? What do your neighbors think about HIV testing?
7. Do the HIV testing services at facilities/community outreaches make you feel like testing for HIV? Why say so?
8. What could be the possible reasons for very few youths testing for HIV in your community?
9. What do you think can be done to increase the number of youths going for HIV testing in this community?
10. Is there anything you think I haven’t asked you about?

**Thanks once again for participating in this study**
