# Supplementary material for: Determinants of testing for HIV among young people in Uganda. A nested, explanatory-sequential study
Source: PLOS Glob Public Health. 2022 Dec 5;2(12):e0000870. doi: 10.1371/journal.pgph.0000870 (PMC10022384; doi:10.1371/journal.pgph.0000870)
Supplement: Attachment — Submitted filename: Response to Reviewers.docx [file pgph.0000870.s003.docx]

Dennis Kalibbala

MUJHU Research Collaboration

P.O. Box 23491, Kampala, Uganda

14 January 2022

Dear Editors

We thank the reviewers for their generous comments on the manuscript (PGPH-D-21-00782). We have edited the manuscript to address their concerns as follows;

| **Comment** | **Response** |
| --- | --- |
| 1.Please provide additional details regarding participant consent. In the ethics statement in the Methods and online submission information, please ensure that you have specified what type of consent you obtained (for instance, written or verbal, and if verbal, how it was documented and witnessed).  If your study included minors, state whether you obtained consent from parents or guardians. | Additional details on consenting added on page 6. Written informed consent was obtained from each participant prior to participation.  Minimum age was 15 years and these assented after consent from their parent/ guardians. |
| 2.In the online submission form, you indicated that "The datasets used and/or analyzed during the current study are available from the corresponding author on reasonable request."  All PLOS journals now require all data underlying the findings described in their manuscript to be freely available to other researchers, either 1. In a public repository, 2. Within the manuscript itself, or 3. Uploaded as supplementary information. | The Data will be freely available to other researchers and will be upload as a supplementary information after being de-identified. |
| 3. State the initials, alongside each funding source, of each author to receive each grant. | Added |
| **Reviewer #1** |  |
| 1. Some of the study participants reported history of ever HIV testing and more than 5% of them were positive. It is essential to discuss the influence of local HIV infection prevalence on the observed results. | We have added a discussion of the influence of the local HIV infections prevalence on the observed results as explained by the young people in the qualitative interviews that some feared retesting for HIV due to self-perceived elevated risk. |
| 2. In Table 1, the characteristics of the study participants should be displayed in subgroups according to rural/urban status according to the used stratified cluster random sampling method. I would suggest to list rural/urban status in this table 3 as well | Data was declared as a survey design dataset on rural and urban since this was a community survey study. Therefore, its effect was adjusted for in the results in table 3. |
| 3. In Table 3, there is a wrong number in "Ever had sexual Intercourse" as "149(87.3)". | Thanks for this observation, this has been corrected to 149(66.5). |
| 4. The identified independent determinants in table 3 mostly showed associations in relatively weak strength. Please discuss the significance of such findings for directing practice. | The study had clustering and the prevalence ratios with clustered robust standard errors. The relatively weak strength still direct practice. |
| **Reviewer #2** |  |
| : Any more info on a) gender, study focuses on these two age groups, but would have provided insight to breakdown by gender as well. I would at least bring findings more into the discussion as likely there are more differences. would show if age, marital status, ever had sex vary by gender. Gender disaggregated data would likely be insightful. b) locality prevalence rates (again by gender would be helpful) | Included the disaggregation in table 1 with chi-square p-values.  Included |
| b) Is there any more insight into knowledge of treatment options, effectiveness of treatment? often helps improve test-seeking | We tried to asses knowledge on treatment options in the qualitatively during probing. They seemed aware but with perceived consequences. |
| c) Any insight into % of people who know someone close to them with HIV, often helps improve test-seeking | From the interviews still we had one of the participants who shared staying with the Auntie living with HIV so she had tested before but still not aware of her status and fearing to re-test. |
| d) ANC testing rates? if facility-based birth, all actually tested? % Facility based? % Who have a child or are pregnant/have pregnant partner? | We did not primarily collect the proportions of ANC and Facility based birth testing, but from table 2 there were 28 participants who were first tested when they had gone for ANC. |
| e) Healthcare interactions regularly lead to testing or offers for testing, especially hospitalization and ANC, but also other clinic interactions? is this the norm? | It is the Norm and from table 2, there were participants who had gone for other services and also tested for HIV. However, this is not a must in private hospitals. |
| Line 102. approx size of parishes. how many residents or households | Added number of residents per parish and a reference |
| Line 108 gender not used in selection? | Gender was used in the selection. This has been corrected |
| Line 178 distance (lower case) | corrected |
| Line 183 Is Table 4 necessary? Given the population of Namayumba is under 20K this would be consider Protected Health Information in general. It is not clear this adds to the document. Would rather just a brief description of ages, geography, gender etc | It describes the participants interviewed. The population of Namutumba is more than 20k and I have included it as well. |
| Line 205 Any information on perceived availability of treatment? know people who are treated? know that treatment is effective. Any impact of knowing someone with HIV? | They were aware that treatment is available and effective. There was some impact of knowing some with HIV (Table 5E). |
| Line 250 Isn't the 95-95-95 that 95% of PLHIV know their diagnosis. It's slightly different than "testing prevalence remains below the UNAIDS target of 95%" | Yes and given that only 80.2% have ever tested, to achieve the target of 95%, 95% of the youth need to test for HIV and also test frequently to remain aware of their status. |
| Line 317 in what way well trained? would just be specific.  Line 328 That is very interesting that many tested unsure of status. | Specified  This was one of the major issues |
| Discussion What reasons do you see for the higher rates here? | The high prevalence can also be attributable to the difference in time and the fact that HIV testing varies considerably across different settings. |
| What recommendations on frequency of testing would you look for? Given high incidence, would seem testing more frequently would be needed to meet 95-95-95. | Yes, testing more frequently to meet the 95-95-95. This has been added. |

Thanks you for your kind considerations.

Yours sincerely,

Mr. Dennis Kalibbala
