# Supplementary material for: Determinants of testing for HIV among young people in Uganda. A nested, explanatory-sequential study
Source: PLOS Glob Public Health. 2022 Dec 5;2(12):e0000870. doi: 10.1371/journal.pgph.0000870 (PMC10022384; doi:10.1371/journal.pgph.0000870)
Supplement: Attachment — Submitted filename: Response to Reviewers.docx [file pgph.0000870.s004.docx]

Dennis Kalibbala

MUJHU Research Collaboration

P.O. Box 23491, Kampala, Uganda

16 May 2022

Dear Editor

We thank the reviewers for their generous comments on the manuscript (PGPH-D-21-00782). We have edited the manuscript to address their concerns as follows;

| **Comment** | **Response** |
| --- | --- |
| 1. Your co-authors, Steven Kiwuwa Mpungu (mkiwuwa@yahoo.com), Bashir Ssuna (sbn144@gmail.com), Happiness Mberesero (happymberesero@gmail.com), Anne Katahoire (annekatahoire@yahoo.co.uk), Mari Armstrong-Hough (mah842@nyu.edu),  Joan N. Kalyango (nakayaga2001@yahoo.com), and Victor Musiime (musiimev@yahoo.co.uk), have not confirmed authorship of the manuscript. We have resent them the authorship confirmation email; however please check that the above email address for them is correct and follow up personally to ensure they confirm. Please note that we cannot pass your manuscript to Production until we have received confirmations from all co-authors. | All co-authors have confirmed authorship |
| 2. Please update your Competing Interests statement. If you have no competing interests to declare, please state: “The authors have declared that no competing interests exist.” | Our competing Interests statement has been updated as advised. |
| 3. Please ensure that you refer to Table 4 in your text as, if accepted, production will need this reference to link the reader to the table. | We have decided to drop table 4 as advised by review 2. |
| 4. Please review your reference list to ensure that it is complete and correct. If you have cited papers that have been retracted, please include the rationale for doing so in the manuscript text, or remove these references and replace them with relevant current references. Any changes to the reference list should be mentioned in the rebuttal letter that accompanies your revised manuscript. If you need to cite a retracted article, indicate the article’s retracted status in the References list and also include a citation and full reference for the retraction notice. | The reference list has been reviewed and is correct. No paper has been retracted. |
| **Reviewer #1** |  |
| The TEXT needs big improvement. For example, two numbers in lines 99-100 were missed | The two numbers have been included and the text reviewed |
| **Reviewer #2** |  |
| Line 42. would rework this sentence. The prevalence testing for HIV was 80.2% It will likely be misread, though is accurate. | This has been revised to “The prevalence of testing for HIV was 80.2%.” |
| Line 64-5, would state year of figure (published in 2016 and I suspect it has increased substantially since then). Would also stress than 95-95-95 requires 95% of those who are HIV+ to know their status. This isn't the same as saying 95% of youth need to be tested. depends on epidemic and risk though certainly everyone knowing their status would lead to this goal. | Thanks for this comment. This has been revised to indicate by 2016 (6). The goal was 90-90-90 by 2020, we did not stress that because this keeps changing. |
| Table 4 is still a concern that shares more info than we need. Ethically this is a concern and I would not include this detailed info here. This is PHI and not clear the participants fully agreed to have this level of info shared. Technically, someone reading this in the area might be able to guess who someone was. We really do not need to precise age so no reason to provide something this identifying and not sure even area they are from is needed. Could just provide percent of participants from each area, age median, range in age and breakdown in gender. | Thanks for this observation, we have decided to drop table 4. |

Thanks, for your kind consideration.

Yours sincerely,

Mr. Dennis Kalibbala
