# Supplementary material for: Determinants of testing for HIV among young people in Uganda. A nested, explanatory-sequential study
Source: PLOS Glob Public Health. 2022 Dec 5;2(12):e0000870. doi: 10.1371/journal.pgph.0000870 (PMC10022384; doi:10.1371/journal.pgph.0000870)
Supplement: Attachment — Submitted filename: Response to Reviewers.docx [file pgph.0000870.s005.docx]

Dennis Kalibbala

MUJHU Research Collaboration

P.O. Box 23491, Kampala, Uganda

09 Oct 2022

Dear Editor

We thank the reviewers for their generous comments on the manuscript (PGPH-D-21-00782). We have edited the manuscript to address their concerns as follows;

| **Comment** | **Response** |
| --- | --- |
| Kindly rephrase the first sentence in the results section of the abstract and in line 187 (page 9) relating to the rate of uptake of HIV testing services to avoid any ambiguity as has been suggested by Review #2. | Corrected to “The prevalence of ever testing was 80.2% (95%CI: 76.9-83.1%) while the prevalence of testing in the last one year was 75.0% (95%CI: 71.1-78.6%).” |
| Review the manuscript for grammatical and editorial errors for better clarity. | Reviewed and corrected |
| **Reviewer #1** |  |
| **Reviewer #2** |  |
| I would still recommend changing, for clarity, the sentence in the abstract that reads "The prevalence testing for HIV was 80.2%." This will be misread as 80% have HIV. | Done |
| **Reviewer #3** |  |
| How does HIV testing prevalence in these young people compare with older persons (>24 years)? What is the estimated testing prevalence in older folks? Wouldn’t most of the identified barriers also apply to older persons, resulting in broad policy implications? | Older persons have more opportunities for testing and are less likely to fear older health workers. for example, those >24 years are more likely to be married and get tested during Antenatal checkups compared to adolescents. |
| Results: Tables 3. Seems longer distance (>10kms from a facility) was associated with a decreased likelihood of testing, but language in lines 196 – 201 suggests otherwise. Could the authors restructure this? | Corrected to “…while those living >10 km from a facility (aPR=0.77, 95%CI: 0.59-0.99) were less likely to test for HIV…” |
| Table 2. Please provide N for each category (Testing N=xx, Not Testing N=yy). | Added |
| Line 235 – 236; the sentence beginning with “For example, one of …” is incomplete | Removed |
| Line 248 – 249. Please check grammar. | Corrected |
| Line 251. Change “introduce” to “introduced”. Line 252, change “attends” to “attended”. | Corrected |
| Line 253 – 254. Sentence beginning with “Young people also …” not clear. Please reword. | Reworded |

Thanks, for your kind consideration.

Yours sincerely,

Mr. Dennis Kalibbala
